# The Impact of Chronic Kidney Disease on In‐Hospital Outcomes in Patients With Acute Respiratory Distress Syndrome

**DOI:** 10.1155/carj/9063636

**Published:** 2026-01-13

**Authors:** Adishwar Rao, Ayesha Anwar, Akriti Agrawal, Asim Kichloo, Jagmeet Singh, Apurwa Karki

**Affiliations:** ^1^ Department of Internal Medicine, Guthrie Robert Packer Hospital, Sayre, Pennsylvania, 18840, USA; ^2^ Department of Medicine and Geriatrics, Texas College of Osteopathic Medicine, University of North Texas Health Science Center, Fort Worth, Texas, 76107, USA; ^3^ Department of Nephrology, Guthrie Robert Packer Hospital, Sayre, Pennsylvania, 18840, USA; ^4^ Department of Pulmonary Critical Care Medicine, Guthrie Robert Packer Hospital, Sayre, Pennsylvania, 18840, USA

**Keywords:** acute respiratory distress syndrome, chronic kidney disease, hospital mortality, outcomes

## Abstract

**Background:**

Acute respiratory distress syndrome (ARDS) is associated with high mortality rates in critically ill patients. Renopulmonary interplay remains crucial in contributing to the outcomes in patients with ARDS. While the role of acute kidney injury has been widely explored in these patients, there remains an unmet need in the literature about the impact of chronic kidney disease (CKD) in these patients.

**Research Question:**

Is there a quantifiable association between CKD and in‐hospital outcomes in patients with ARDS?

**Study Design and Methods:**

We utilized a retrospective study design to compare descriptive statistics and outcomes in patients with ARDS with or without CKD. Pearson’s chi‐square test was used to compare categorical variables, while the Wilcoxon rank sum test was used for continuous variables. We also performed multivariate logistic regression analyses for each outcome and adjusted for demographics and comorbidities. Lastly, we conducted a sensitivity analysis using propensity score–matched outcomes between these groups.

**Results:**

Among 479,450 patients with ARDS, 17.6% also had CKD, while 82.4% did not. Patients with ARDS and CKD were older (median age: 71 years vs. 60 years, *p* < 0.001) and comprised a greater proportion of males (59.4% vs. 55.9%, *p* < 0.001). CKD was associated with increased odds of in‐hospital mortality (adjusted odds ratio [aOR] 1.29, *p* < 0.001), acute heart failure (aOR 1.26, *p* < 0.001), ventricular arrhythmias (aOR 1.16, *p* < 0.001), cardiogenic shock (aOR 1.10, *p* = 0.044), major adverse cardiovascular events (aOR 1.29, *p* < 0.001), and length of stay ≥ 7 days (aOR 1.05, *p* = 0.033).

**Interpretation:**

Our study provides insights into the magnitude of impact renal diseases may have on the outcomes of patients with ARDS. Further prospective studies are warranted to establish more substantial epidemiological evidence of this relationship to tailor the management of such patients.

## 1. Introduction

Acute respiratory distress syndrome (ARDS) is a syndromic manifestation of acute hypoxemic respiratory failure (AHRF) secondary to inflammatory pulmonary pathology after the exclusion of cardiogenic pulmonary edema [1]. The definition of ARDS has been refined multiple times due to ever‐evolving clinical knowledge and technological techniques, including pulse oximetric methods and oxygen delivery strategies [2–7]. The Berlin definition of ARDS remains the most referred definition of ARDS [6]. However, during the coronavirus disease 2019 (COVID‐19) pandemic, the definition’s criterion of ARDS involving a minimum positive end‐expiratory pressure of 5 cm of water via invasive or noninvasive ventilation was often not satisfied as AHRF was frequently managed with high flow nasal oxygen, and the definition was severely limited in identifying patients in resource‐limited settings. This also led to the inception of the Kigali modification, leading to a proposed new global definition of ARDS that addresses these shortcomings [8].

The renopulmonary interactions regarding acute kidney injury (AKI) and its interaction with the pathophysiology of ARDS have been extensively studied. Multiple studies have delved into the impact of AKI in patients with ARDS concerning mortality and in‐hospital outcomes [9–11]. Interestingly, there appear to be no studies exploring the relationship between underlying chronic kidney disease (CKD) and in‐hospital outcomes in patients diagnosed with ARDS, despite the prevalence of CKD being approximately 14% in the United States (US) population, per the National Institute of Health’s (NIH’s) United States Renal Data System‐Annual Data Report (USRDS‐ADR) [12]. Therefore, in this study, we query an extensive national US healthcare database to understand the potential impact of CKD on in‐hospital mortality and outcomes in patients diagnosed with ARDS.

## 2. Methods

### 2.1. Data Source and Cohort Selection

We conducted a cross‐sectional study using the National Inpatient Sample (NIS) database provided by the Agency of Healthcare Quality and Research (AHRQ) [13]. NIS is the largest inpatient database in the US and has been developed as a part of the Healthcare Cost and Utilization Project (HCUP). It consists of data on inpatient healthcare utilization, including diagnoses, treatments, and hospital metrics [13]. We queried the NIS from 2016 to 2021 for hospitalizations related to all‐cause ARDS using the International Classification of Diseases‐10 Clinical Modification (ICD‐10 CM) code J80 [14]. We then divided this sample into two groups based on the presence or absence of CKD using ICD‐10 CM codes. We initially included all CKD patients and excluded those with end‐stage renal disease/Group 5 CKD. A list of ICD codes used in the study has been provided in the Supporting Information.

### 2.2. Study Variables and Outcomes

Descriptive statistics of the baseline study sample include demographic variables, hospital metrics, and comorbidities. Demographics comprise age, sex assigned at birth, and race/ethnicity. Sex assigned at birth is either male or female, while race/ethnicity included White Americans, African Americans, Hispanics, Asians or Pacific Islanders, Native Americans, and others, based on the structure of data provided in the national database. Hospital metrics included the primary financial sponsor for the hospitalization, consisting of Medicare, Medicaid, private insurance, self‐pay, absence of charges, or other sources, and the hospital’s region and location/teaching status.

We included multiple comorbidities such as cardiovascular comorbidities and risk factors, prior cardiac procedures, pulmonary diseases, and other diseases in the baseline assessment of our sample. Cardiovascular comorbidities included hypertension, hyperlipidemia, chronic heart failure, atrial fibrillation/flutter, stress cardiomyopathy, previous myocardial infarction (MI), obesity, and smoking/tobacco use, while history of percutaneous coronary intervention (PCI), coronary artery bypass grafting (CABG), and pacemaker/defibrillator implantation was included in prior cardiac procedures. Furthermore, pulmonary diseases included chronic obstructive pulmonary disease (COPD), obstructive sleep apnea (OSA), pulmonary hypertension (PH), and COVID‐19. Other comorbidities included in the study were liver disease, diabetes mellitus (DM), hypothyroidism, nutritional anemia, and history of stroke.

The primary outcome of the study was in‐hospital mortality, while the secondary outcomes included clinical events such as ST elevated MI (STEMI), acute heart failure (AHF), ventricular arrhythmias, cardiogenic shock (CS), and major adverse cardiac events (MACEs) and hospital metrics such as length of stay of 7 days or more. Any patient who suffered from STEMI, CS, or experienced in‐hospital mortality was considered to have a MACE.

### 2.3. Statistical Analysis

We assessed the normality of the data distribution using histograms and QQ plots. We used the Pearson chi‐square test to compare categorical variables and the Wilcoxon rank sum test to compare continuous variables between the two study groups. Categorical variables were reported as absolute counts with their respective percentages, while continuous variables were reported as medians with their respective interquartile ranges (IQRs). We conducted a multivariate logistic regression analysis to compute adjusted odds ratios (aORs) of outcomes with their 95% confidence intervals (95% CIs) and *p* values while adjusting for confounding factors. We also conducted a sensitivity analysis, using 1:1 nearest neighbor propensity score matching with no replacement, to strengthen our statistical inferences. We used the DISCWT variable provided in the dataset to calculate weighted estimates for all variables in the study. A *p* value < 0.05 was considered to be statistically significant for the study. We used Stata 18, provided by Stata Corp LLC, TX, USA, to conduct all statistical analyses used in this study. We used Microsoft Excel to design graphic illustrations relevant to this study.

### 2.4. Institutional Review Board (IRB) Approval

Our study was exempt from IRB approval due to the publicly available nature of the NIS database, which is extensively deidentified to prevent disclosure of patient‐specific information. Moreover, to comply with the publication guidelines issued by AHRQ‐HCUP, we have not reported descriptive statistics and results comprising less than 11 patients in the study.

## 3. Results

### 3.1. Descriptive Statistics of the Sample

We assessed descriptive statistics of a weighted sample of 479,450 ARDS patients, comparing those with and without CKD. 17.6% (84,300) of ARDS patients also had CKD, while 82.4% (395,150) did not (Table 1). ARDS patients with CKD were older and had a higher proportion of males compared to those without CKD. There were significant differences in the racial distribution of ARDS patients with CKD compared to those without CKD (all *p* < 0.001). Moreover, ARDS patients with CKD had a significantly higher prevalence of hyperlipidemia, hypertension, chronic heart failure, atrial fibrillation/flutter, history of MI, prior PCI, prior CABG, preexisting pacemaker or defibrillator, COPD, OSA, PH, history of stroke, DM, hypothyroidism, and nutritional anemia compared to those without CKD (all *p* < 0.05). On the contrary, ARDS patients with CKD had a lower prevalence of smokers/tobacco users, stress cardiomyopathy, liver disease, and COVID‐19 than those without CKD (all *p* < 0.05).

**Table 1:** Descriptive statistics of ARDS patients with and without CKD.

	ARDS with CKD	ARDS without CKD	p
*N*	84,300 (17.6%)	395,150 (82.4%)	
Sex assigned at birth			
Male	50,040 (59.4%)	220,800 (55.9%)	< 0.001
Female	34,260 (40.6%)	174,350 (44.1%)	
Age (y)	71 (61‐78)	60 (48‐70)	< 0.001
Race			
White	49,135 (60.1%)	227,080 (59.8%)	< 0.001
Black	16,790 (20.5%)	50,770 (13.4%)	
Hispanic	10,125 (12.4%)	69,715 (18.4%)	
Asian or Pacific Islander	2,860 (3.5%)	12,515 (3.3%)	
Native American	695 (0.9%)	5,330 (1.4%)	
Other	2,130 (2.6%)	14,475 (3.8%)	
Primary insurance			
Medicare	56,985 (67.7%)	159,800 (40.5%)	< 0.001
Medicaid	8,690 (10.3%)	75,440 (19.1%)	
Private insurance	14,550 (17.3%)	122,555 (31.1%)	
Self‐pay	1,625 (1.9%)	18,500 (4.7%)	
No charge	105 (0.1%)	1,035 (0.3%)	
Other	2,230 (2.6%)	16,905 (4.3%)	
Region of hospital			
Northeast	14,295 (17.0%)	66,220 (16.8%)	< 0.001
Midwest	21,055 (25.0%)	83,400 (21.1%)	
South	32,970 (39.1%)	154,635 (39.1%)	
West	15,980 (19.0%)	90,895 (23.0%)	
Location/teaching status of hospital			
Rural	6,820 (8.1%)	32,500 (8.2%)	0.289
Urban nonteaching	14,795 (17.6%)	67,030 (17.0%)	
Urban teaching	62,685 (74.4%)	295,620 (74.8%)	
Bed size of the hospital			
Small	17,100 (20.3%)	77,460 (19.6%)	0.011
Medium	23,395 (27.8%)	106,245 (26.9%)	
Large	43,805 (52.0%)	211,445 (53.5%)	
Comorbidities			
Hyperlipidemia	39,705 (47.1%)	121,115 (30.7%)	< 0.001
Hypertension	76,135 (90.3%)	206,560 (52.3%)	< 0.001
Chronic heart failure	38,955 (46.2%)	69,660 (17.6%)	< 0.001
Atrial fibrillation/flutter	27,620 (32.8%)	75,340 (19.1%)	< 0.001
Stress cardiomyopathy	260 (0.3%)	2,465 (0.6%)	< 0.001
Prior MI	5,770 (6.8%)	11,265 (2.9%)	< 0.001
Prior PCI	4,470 (5.3%)	10,400 (2.6%)	< 0.001
Prior CABG	6,105 (7.2%)	11,675 (3.0%)	< 0.001
Prior pacemaker or defibrillator	3,950 (4.7%)	6,465 (1.6%)	< 0.001
Obesity	28,510 (33.8%)	135,595 (34.3%)	0.240
Smoker/tobacco user	22,650 (26.9%)	110,895 (28.1%)	0.003
COPD	21,535 (25.5%)	65,945 (16.7%)	< 0.001
Obstructive sleep apnea	10,870 (12.9%)	35,925 (9.1%)	< 0.001
Pulmonary hypertension	7,325 (8.7%)	19,080 (4.8%)	< 0.001
Prior stroke	7,505 (8.9%)	17,980 (4.6%)	< 0.001
Liver disease	11,375 (13.5%)	62,175 (15.7%)	< 0.001
Diabetes mellitus	46,960 (55.7%)	130,765 (33.1%)	< 0.001
Hypothyroidism	12,285 (14.6%)	39,950 (10.1%)	< 0.001
Nutritional anemia	6,270 (7.4%)	21,580 (5.5%)	< 0.001
COVID‐19	45,470 (53.9%)	224,280 (56.8%)	< 0.001

Insurance utilization was significantly different between ARDS patients with and without CKD. While Medicare, private insurance, and Medicaid were the most common primary payers among both groups, the group with CKD had higher utilization of Medicare but lower utilization of Medicaid and private insurance than the group without CKD. Regional distribution of hospitals caring for these patients also varied between the ARDS with CKD and ARDS without CKD groups (both *p* < 0.001).

### 3.2. Outcomes

**Figure 1:**
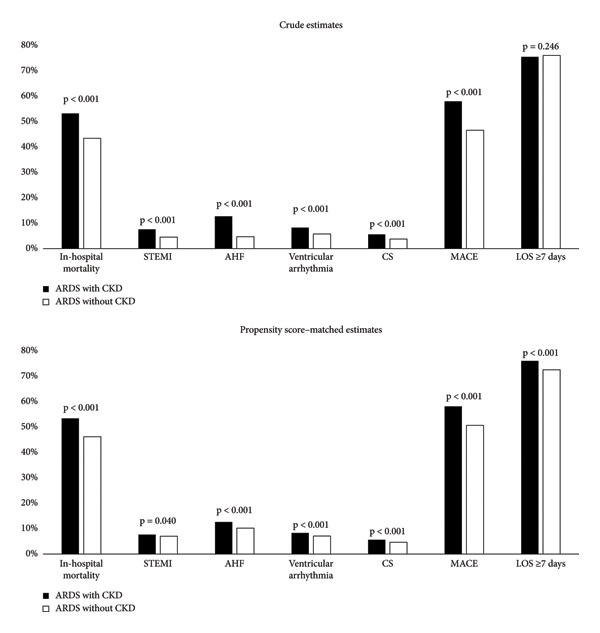
Crude and propensity score–matched outcomes in ARDS patients with and without CKD. STEMI, ST segment elevated myocardial infarction; AHF, acute heart failure; CS, cardiogenic shock; MACE, major adverse cardiac event; LOS, length of stay; *p*, *p* value.

Comparison of primary and secondary outcomes of the study between ARDS patients with and without CKD revealed that the patients with CKD had a higher prevalence of in‐hospital mortality (52.9% vs. 43.2%, *p* < 0.001), STEMI (7.5% vs. 4.5%, *p* < 0.001), AHF (12.6% vs. 4.6%, *p* < 0.001), ventricular arrhythmia (8.2% vs. 5.7%, *p* < 0.001), CS (5.5% vs. 3.7%, *p* < 0.001), and MACE (57.6% vs. 46.3%, *p* < 0.001) than those without CKD (Figure 1). There was no statistically significant difference in the proportion of patients hospitalized for ≥ 7 days between the two groups (75.1% vs. 75.6%, *p* = 0.246). Additionally, in ARDS patients with a STEMI during the hospitalization, rates of undergoing PCI were similar between patients with and without CKD (13.3% vs. 13.6%, *p* = 0.797).

**Figure 2:**
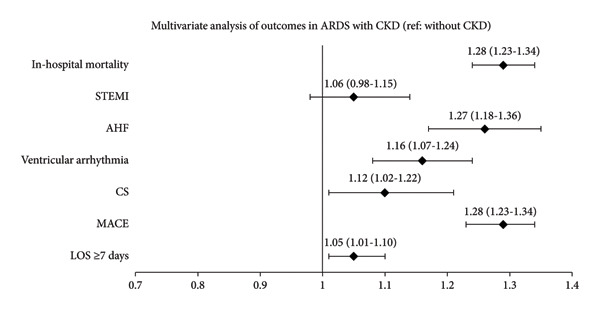
Forest plot from a multivariate analysis in ARDS patients with and without CKD. Ref, reference; STEMI, ST segment elevated myocardial infarction; AHF, acute heart failure; CS, cardiogenic shock; MACE, major adverse cardiac event; LOS, length of stay; data reported as aOR (95% CI).

Results from a multivariate analysis using logistic regression while adjusting for demographics and comorbidities suggested that ARDS patients with CKD had higher odds of in‐hospital mortality (aOR 1.28, *p* < 0.001), AHF (aOR 1.27, *p* < 0.001), ventricular arrhythmia (aOR 1.16, *p* < 0.001), CS (aOR 1.12, *p* = 0.022), and MACE (aOR 1.28, *p* < 0.001) than patients without CKD (Figure 2). Moreover, ARDS patients with CKD also had higher odds of requiring hospitalization for 7 days or more compared to those without CKD (aOR 1.05, *p* = 0.046).

Upon conducting a propensity‐matched analysis using demographics and comorbidities to calculate propensity scores and adjusting for these variables, it was revealed that patients with ARDS and CKD had higher prevalence of in‐hospital mortality (52.8% vs. 45.7%, *p* < 0.001), STEMI (7.5% vs. 6.9%, *p* = 0.040), AHF (12.5% vs. 10.1%, *p* < 0.001), ventricular arrhythmia (8.2% vs. 7%, *p* < 0.001), CS (5.5% vs. 4.6%, *p* < 0.001), and MACE (57.5% vs. 50.1%, *p* < 0.001). The proportion of patients requiring hospitalization for seven or more days was also higher in ARDS patients with CKD compared to those without CKD (75.2% vs. 71.8%, *p* < 0.001).

## 4. Discussion

Our study highlights the significance of the kidney–lung relationship and its effect on mortality in the in‐hospital setting. We included a weighted sample of 479,450 patients with ARDS, among which 84,300 (17.6%) had CKD while 395,150 (82.4%) did not. In‐hospital outcomes were generally worse in patients with CKD compared to those without CKD, including greater mortality, AHF, ventricular arrhythmias, CS, MACE, and LOS ≥ 7 days.

The adverse prognostic impact of AKI in ARDS patients has been studied previously. However, the literature on the effects of CKD on outcomes in ARDS is limited. A retrospective cohort study performed in the past showed that CKD was associated with increased odds of mortality in mechanically ventilated ARDS patients (OR 1.75, 95% CI 1.22–2.50, *p* = 0.02) [15]. Moreover, in a study by Kao et al., a high Charlson Comorbidity Index was associated with greater short‐term mortality [16]. While the LUNG SAFE study reported that the mortality in ARDS patients increases from approximately 40%–60% in the presence of AKI, our study also showed a similar mortality of nearly 52.8% in ARDS patients with CKD [17]. Notably, the mortality rate in our study was comparable to that seen in patients with ARDS and AKI, as noted in a secondary data analysis of the ARDSNet trial [18]. Interestingly, a study by Hendrickson et al. demonstrated the association between the level of cystatin C, which is a marker of CKD, and 60‐day mortality in patients with ARDS [19]. Similarly, another study demonstrated β2‐microglobulin, an endogenous marker for estimation of GFR, as an independent predictor for 28‐day mortality in ARDS patients [20]. The evidence obtained from our study points toward a higher prevalence of in‐hospital mortality in ARDS patients with CKD, even after adjusting for demographics and multiple comorbidities.

Our study also demonstrates a greater prevalence of poor cardiovascular outcomes in patients with ARDS and CKD. Renopulmonary interactions may impact fluid homeostasis, hypoxemia, and systemic inflammation in the body, which can result in impaired blood pressure control, worsening of preexisting heart failure, and numerous arrhythmias [21]. Bromley et al. analyzed non‐COVID‐19 ARDS deaths and suggested that while multiple organ failure is a leading cause of death in these patients, pulmonary failure (42%) and cardiac failure (37%) were the most common causes of death in the absence of a predetermined category for multiple organ failure [22]. Additionally, a meta‐analysis by Jayasimhan et al. demonstrated the association of elevated cardiac markers with higher mortality in ARDS [23].

The association between CKD and cardiovascular disease has also been shown extensively in the literature, and it is well understood that cardiovascular pathophysiology remains the leading cause of mortality in patients with CKD, accounting for 40%–45% of deaths in patients with advanced CKD [24]. The presence of heart failure as a factor associated with the development of kidney injury in ARDS has also been studied previously [11]. Although outcomes of critical illness requiring ICU admission have been previously studied in patients on chronic hemodialysis, dialysis‐induced predisposition to cardiovascular morbidity and mortality is a well‐known phenomenon [25, 26]. Therefore, we excluded patients receiving chronic hemodialysis to limit dialysis‐related confounding in our study. CKD was still associated with increased odds of AHF, ventricular arrhythmia, CS, and MACE in ARDS [27]. However, lacunae continue to exist in the availability of actual world clinical data for cardiovascular disease in ARDS patients with CKD, and even though statistically adjusted for, the baseline differences in demographics and comorbidities may account for some degree of worse outcomes in the group with CKD in our study.

In‐hospital stay duration may significantly contribute to hospital‐acquired complications, including infectious and iatrogenic complications [28]. We compared the proportion of ARDS patients requiring an extended hospital stay, ≥ 7 days, based on whether they had concomitant CKD after matching the groups on propensity scores. A significantly greater proportion of ARDS patients with CKD required a longer hospital stay compared to those without CKD, prompting the contribution of CKD toward the extended hospital stay in a greater proportion of patients. It has been previously demonstrated that the development of AKI in ARDS may lead to increased ventilator days and duration of weaning [29]. However, the literature regarding CKD is severely limited. In a retrospective cohort study using the NIS database conducted between 2016 and 2018, the length of stay was higher in ARDS patients with preexisting CKD. However, this difference was not statistically significant (incidence rate ratio 1.04, 95% CI 0.9–1.19, *p* = 0.62) [15]. Therefore, our study is the first to demonstrate a significant association between CKD and extended hospital stays in patients with ARDS.

### 4.1. Study Limitations

As with any research project, our study is subject to certain limitations, mainly due to study design limitations, database characteristics, selective availability of clinical data, lack of pulmonary artery catheterization data, and unknown confounders. Since both ARDS and the evaluated outcomes (such as STEMI and CS) represent acute events, determining their chronological sequence is essential for causal inference; however, due to the cross‐sectional nature of the database, it was not possible to ascertain whether these outcomes occurred during the course of ARDS or following its resolution, as the dataset only captures that both events occurred within the same hospitalization without specifying their timing. Moreover, the clinical outcomes may vary significantly based on the severity of ARDS, which the database does not adequately describe. Similarly, the current study’s correlation between early versus later stages of CKD and ARDS is limited. The results may be inherently biased due to the underlying severity of CKD. We used statistical methods such as multivariate logistic regression and propensity score matching to address known confounding factors affecting our study variables; however, it is challenging to assess the effects of unknown confounders, which may have influenced our results. Even though rigorously screened for errors, the database is an administrative database and is not immune to coding and typographical errors. Additionally, the ICD codes may not be able to differentiate between subtypes of shock with absolute precision, particularly in the absence of hemodynamic or laboratory data, and there may be potential for misclassifications, especially in a complex critical illness such as ARDS. Lastly, the NIS lacks data on right heart catheterization; if available, it may assist in eliminating further confounding and providing a more accurate representation of the hemodynamic status in these patients. However, it has not been demonstrated to alter the overall prognosis significantly and is not routinely emphasized in patients with ARDS [30]. Despite all these shortcomings, the study’s novel research question and significant sample size contribute to its strength.

## 5. Conclusion

ARDS is a critical illness that is associated with a significant increase in mortality. In these patients, the coexistence of CKD will likely worsen clinical outcomes. We discussed the impact CKD may have on in‐hospital outcomes in patients diagnosed with ARDS. CKD was generally associated with worse in‐hospital outcomes in patients with ARDS. There continues to exist a sizeable need for further research into this arena, given the ever‐evolving landscape of ARDS diagnosis and management and the advancements in the prevention and management of CKD.

## Conflicts of Interest

The authors declare no conflicts of interest.

## Funding

The authors received no specific funding for this work.

## Supporting Information

Table S1: crude and propensity‐matched outcomes of ARDS patients with CKD and without CKD.

Table S2: multivariate analyses of outcomes in ARDS patients with CKD with ARDS patients without CKD as reference.

Table S3: list of ICD‐10 CM and ICD‐10 PCS codes used for identification of the cohort, comorbidities, and outcomes in the study.

## Supporting information


**Supporting Information** Additional supporting information can be found online in the Supporting Information section.

## Data Availability

The National Inpatient Sample data used to support the findings of this study have not been made available because they are publicly sourced through the Agency of Healthcare Research and Quality under the ambit of the Healthcare Cost and Utilization Project and can be requested only through them.
