# Supplementary material for: The Impact of Chronic Kidney Disease on In‐Hospital Outcomes in Patients With Acute Respiratory Distress Syndrome
Source: Can Respir J. 2026 Jan 13;2026:9063636. doi: 10.1155/carj/9063636 (PMC12797150; doi:10.1155/carj/9063636)
Supplement: Supplementary file 1 — Supporting Information Additional supporting information can be found online in the Supporting Information section. [file CARJ-2026-9063636-s001.docx]

**Supplemental File**

**Title**: The Impact of Chronic Kidney Disease On In-Hospital Outcomes in Patients With Acute Respiratory Distress Syndrome

**Authors:** Adishwar Rao MD, MPH^1^, Ayesha Anwar MD^1^, Akriti Agrawal MD, MPH^1^, Asim Kichloo MD, FACP^2^, Jagmeet Singh MD, FASN, Apurwa Karki MD, FCCP^3^

**Affiliations**:

^1^Department of Internal Medicine, Guthrie Robert Packer Hospital, Sayre, PA, 18840, USA

^2^Department of Medicine and Geriatrics, Texas College of Osteopathic Medicine, University of North Texas Health Science Center, Fort Worth, TX, 76107, USA

^3^Department of Nephrology, Guthrie Robert Packer Hospital, Sayre, PA, 18840, USA

^4^Department of Pulmonary Critical Care Medicine, Guthrie Robert Packer Hospital, Sayre, PA, 18840, USA

**Supplemental Table 1: Crude and propensity-matched outcomes of ARDS patients with CKD and without CKD**

|  | **Crude Outcomes** | | | **Propensity Score Matched** | | |
| --- | --- | --- | --- | --- | --- | --- |
|  | **ARDS + CKD** | **ARDS only** | **p** | **ARDS + CKD** | **ARDS only** | **p** |
| N | 84,300 (17.6%) | 395,150 (82.4%) |  | 16,347 (50.0%) | 16,347 (50.0%) |  |
| In-Hospital Mortality | 44,585 (52.9%) | 170,660 (43.2%) | **<0.001** | 8,628 (52.8%) | 7,462 (45.7%) | **<0.001** |
| ST Elevated MI | 6,360 (7.5%) | 17,645 (4.5%) | **<0.001** | 1,226 (7.5%) | 1,130 (6.9%) | **0.040** |
| Acute Heart Failure | 10,610 (12.6%) | 18,045 (4.6%) | **<0.001** | 2,047 (12.5%) | 1,654 (10.1%) | **<0.001** |
| Ventricular Arrhythmia | 6,930 (8.2%) | 22,480 (5.7%) | **<0.001** | 1,339 (8.2%) | 1,138 (7.0%) | **<0.001** |
| Cardiogenic Shock | 4,645 (5.5%) | 14,700 (3.7%) | **<0.001** | 894 (5.5%) | 754 (4.6%) | **<0.001** |
| MACE | 48,550 (57.6%) | 182,945 (46.3%) | **<0.001** | 9,395 (57.5%) | 8,182 (50.1%) | **<0.001** |
| LOS ≥7 days | 63,335 (75.1%) | 298,645 (75.6%) | 0.246 | 12,291 (75.2%) | 11,751 (71.8%) | **<0.001** |

**Supplemental Table 2: Multivariate analyses of outcomes in ARDS patients with CKD with ARDS patients without CKD as reference**

|  | **aOR (95% CI)** | **p** |
| --- | --- | --- |
| In-Hospital Mortality | 1.28 (1.23-1.34) | **<0.001** |
| ST Elevated MI | 1.06 (0.98-1.15) | 0.122 |
| Acute Heart Failure | 1.27 (1.18-1.36) | **<0.001** |
| Ventricular Arrhythmia | 1.16 (1.07-1.24) | **<0.001** |
| Cardiogenic Shock | 1.12 (1.02-1.22) | **0.022** |
| MACE | 1.28 (1.23-1.34) | **<0.001** |
| LOS ≥7 days | 1.05 (1.01-1.10) | **0.046** |

**Supplemental Table 3: List of ICD-10 CM and ICD-10 PCS codes used for identification of the cohort, comorbidities, and outcomes in the study**

| **Variable** | **ICD-10 CM/PCS codes** |
| --- | --- |
| ARDS | J80 |
| CKD | N181 N182 N183 N184 N189 I129 I130 I1310  (Excluded dialysis dependent patients: N185 N186 I120 I1311 I132 Z992) |
| Hyperlipidemia | E785 E7800 E781 E782 E783 E784 E7849 E780 E7800 E7801 |
| Hypertension | I10 I119 I150 I151 I152 I158 I159 I160 I161 I169 I110 I119 I120 I129 I130 I1310 I1311 I132 |
| Chronic Heart Failure | I501 I5020 I5021 I5022 I5023 I5030 I5031 I5032 I5033 I5040 I5041 I5042 I5043 I50810 I50811 I50812 I50813 I50814 I5082 I5083 I5084 I5089 I509 |
| Atrial Fibrillation/Flutter | I480 I481 I4811 I4819 I482 I4820 I4821 I483 I484 I4891 I4892 |
| Stress Cardiomyopathy | I5181 |
| Prior MI | I252 |
| Prior PCI | Z955 |
| Prior CABG | I252 I25700 I25701 I25708 I25709 I25710 I25711 I25718 I25719 I25720 I25721 I25728 I25729 I25730 I25731 I25738 I25739 I25790 I25791 I25798 I25799 I25810 |
| Prior Pacemaker or Defibrillator | Z950 Z95810 |
| Obesity | E6601 E6609 E661 E662 E668 E669 Z6830 Z6831 Z6832 Z6833 Z6834 Z6835 Z6836 Z6837 Z6838 Z6839 Z6841 Z6842 Z6843 Z6844 Z6845 O99210 O99211 O99212 O99213 O99214 O99215 |
| Smoker/Tobacco user | Z720 O99330 O99331 O99333 O99334 O99335 F17200 F17201 F17203 F17208 F17209 F17210 F17211 F17213 F17218 F17219 F17220 F17221 F17223 F17228 F17229 F17290 F17291 F17293 F17298 F17299 Z87891 |
| COPD | J410 J411 J418 J42 J430 J431 J432 J438 J439 J440 J441 J449 |
| Obstructive Sleep Apnea | G4733 |
| Pulmonary Hypertension | I270 I272 I2720 I2721 I2722 I2723 I2724 I2729 |
| Prior Stroke | Z8673 I6930 I6931 I69310 I69311 I69312 I69313 I69314 I69315 I69318 I69319 I69320 I69321 I69322 I69323 I69328 I69331 I69332 I69333 I69334 I69339 I69341 I69342 I69343 I69344 I69349 I69351 I69352 I69353 I69354 I69359 I69361 I69362 I69363 I69364 I69365 I69369 I69390 I69391 I69392 I69393 I69398 |
| Liver Disease | B180 B181 B182 B188 B189 K700 K7010 K7011 K702 K7030 K7031 K7040 K7041 K709 K710 K7110 K7111 K712 K713 K714 K7150 K7151 K716 K717 K718 K719 K7200 K7201 K7210 K7211 K7290 K7291 K730 K731 K732 K738 K739 K740 K7400 K7401 K7402 K741 K742 K743 K744 K745 K7460 K7469 K750 K751 K752 K753 K754 K7581 K7589 K759 K760 K761 K762 K763 K764 K765 K766 K767 K7681 K7689 K769 K77 |
| Diabetes Mellitus | E1010 E1011 E1021 E1022 E1029 E10311 E10319 E10321 E103211 E103212 E103213 E103219 E10329 E103291 E103292 E103293 E103299 E10331 E103311 E103312 E103313 E103319 E10339 E103391 E103392 E103393 E103399 E10341 E103411 E103412 E103413 E103419 E10349 E103491 E103492 E103493 E103499 E10351 E103511 E103512 E103513 E103519 E103521 E103522 E103523 E103529 E103531 E103532 E103533 E103539 E103541 E103542 E103543 E103549 E103551 E103552 E103553 E103559 E10359 E103591 E103592 E103593 E103599 E1036 E1037X1 E1037X2 E1037X3 E1037X9 E1039 E1040 E1041 E1042 E1043 E1044 E1049 E1051 E1052 E1059 E10610 E10618 E10620 E10621 E10622 E10628 E10630 E10638 E10641 E10649 E1065 E1069 E108 E109 E1100 E1101 E1110 E1111 E1121 E1122 E1129 E11311 E11319 E11321 E113211 E113212 E113213 E113219 E11329 E113291 E113292 E113293 E113299 E11331 E113311 E113312 E113313 E113319 E11339 E113391 E113392 E113393 E113399 E11341 E113411 E113412 E113413 E113419 E11349 E113491 E113492 E113493 E113499 E11351 E113511 E113512 E113513 E113519 E113521 E113522 E113523 E113529 E113531 E113532 E113533 E113539 E113541 E113542 E113543 E113549 E113551 E113552 E113553 E113559 E11359 E113591 E113592 E113593 E113599 E1136 E1137X1 E1137X2 E1137X3 E1137X9 E1139 E1140 E1141 E1142 E1143 E1144 E1149 E1151 E1152 E1159 E11610 E11618 E11620 E11621 E11622 E11628 E11630 E11638 E11641 E11649 E1165 E1169 E118 E119 E1300 E1301 E1310 E1311 E1321 E1322 E1329 E13311 E13319 E13321 E133211 E133212 E133213 E133219 E13329 E133291 E133292 E133293 E133299 E13331 E133311 E133312 E133313 E133319 E13339 E133391 E133392 E133393 E133399 E13341 E133411 E133412 E133413 E133419 E13349 E133491 E133492 E133493 E133499 E13351 E133511 E133512 E133513 E133519 E133521 E133522 E133523 E133529 E133531 E133532 E133533 E133539 E133541 E133542 E133543 E133549 E133551 E133552 E133553 E133559 E13359 E133591 E133592 E133593 E133599 E1336 E1337X1 E1337X2 E1337X3 E1337X9 E1339 E1340 E1341 E1342 E1343 E1344 E1349 E1351 E1352 E1359 E13610 E13618 E13620 E13621 E13622 E13628 E13630 E13638 E13641 E13649 E1365 E1369 E138 E139 |
| Hypothyroidism | E030 E031 E032 E033 E034 E035 E038 E039 |
| Nutritional Anemia | D500 D501 D508 D509 D510 D511 D512 D513 D518 D519 D520 D528 D529 D521 D530 D531 D532 D538 D539 |
| COVID-19 | U071 |
| ST Elevated MI | I2101 I2102 I2109 I2111 I2119 I2121 I2129 I213 I214 |
| PCI | 0270346 027034Z 0270356 027035Z 0270366 027036Z 0270376 027037Z 02703D6 02703DZ 02703E6 02703EZ 02703F6 02703FZ 02703G6 02703GZ 02703T6 02703TZ 02703Z6 02703ZZ 0271346 027134Z 0271356 027135Z 0271366 027136Z 0271376 027137Z 02713D6 02713DZ 02713E6 02713EZ 02713F6 02713FZ 02713G6 02713GZ 02713T6 02713TZ 02713Z6 02713ZZ 0272346 027234Z 0272356 027235Z 0272366 027236Z 0272376 027237Z 02723D6 02723DZ 02723E6 02723EZ 02723F6 02723FZ 02723G6 02723GZ 02723T6 02723TZ 02723Z6 02723ZZ 0273346 027334Z 0273356 027335Z 0273366 027336Z 0273376 027337Z 02733D6 02733DZ 02733E6 02733EZ 02733F6 02733FZ 02733G6 02733GZ 02733T6 02733TZ 02733Z6 02733ZZ |
| Acute Heart Failure | I5021 I5023 I5041 I5043 I50811 I50813 |
| Ventricular Arrhythmia | I470 I472 I4901 I4902 |
| Cardiogenic Shock | R570 |

*ARDS, acute respiratory distress syndrome, CKD, chronic kidney disease, MI, myocardial infarction, PCI, percutaneous coronary intervention, CABG, coronary artery bypass graft, COPD, chronic obstructive pulmonary disease, COVID-19, coronavirus disease 2019, ST Elevated MI, ST-segment elevated myocardial infarction*
